# Combined effect of pulse density and grid cell size on predicting and mapping aboveground carbon in fast-growing Eucalyptus forest plantation using airborne LiDAR data

**DOI:** 10.1186/s13021-017-0081-1

**Published:** 2017-06-07

**Authors:** Carlos Alberto Silva, Andrew Thomas Hudak, Carine Klauberg, Lee Alexandre Vierling, Carlos Gonzalez-Benecke, Samuel de Padua Chaves Carvalho, Luiz Carlos Estraviz Rodriguez, Adrián Cardil

**Affiliations:** 1 Department of Natural Resources and Society, College of Natural Resources, University of Idaho, (UI), 875 Perimeter Drive, Moscow, ID 83843 USA; 2 US Forest Service (USDA), Rocky Mountain Research Station, RMRS, 1221 South Main Street, Moscow, ID 83843 USA; 3 Department of Forest Engineering, Oregon State University, 269 Peavy Hall, Corvallis, OR 97331 USA; 4 College of Forestry, Federal University of Mato Grosso, Av. Fernando Correa da Costa, 2367, Boa Esperança, Cuiabá, MT 78060-900 Brazil; 5 Department of Forest Sciences, College of Agriculture Luiz de Queiroz (ESALQ), University of Sao Paulo (USP), Av. Pádua Dias, 11, Piracicaba, SP 13418-900 Brazil; 6 Tecnosylva, Parque Tecnológico de León, 24009 León, Spain

**Keywords:** Carbon modeling, Remote sensing, Modeling, Forest inventory, Random forest

## Abstract

**Background:**

LiDAR remote sensing is a rapidly evolving technology for quantifying a variety of forest attributes, including aboveground carbon (AGC). Pulse density influences the acquisition cost of LiDAR, and grid cell size influences AGC prediction using plot-based methods; however, little work has evaluated the effects of LiDAR pulse density and cell size for predicting and mapping AGC in fast-growing Eucalyptus forest plantations. The aim of this study was to evaluate the effect of LiDAR pulse density and grid cell size on AGC prediction accuracy at plot and stand-levels using airborne LiDAR and field data. We used the Random Forest (RF) machine learning algorithm to model AGC using LiDAR-derived metrics from LiDAR collections of 5 and 10 pulses m^−2^ (RF5 and RF10) and grid cell sizes of 5, 10, 15 and 20 m.

**Results:**

The results show that LiDAR pulse density of 5 pulses m^−2^ provides metrics with similar prediction accuracy for AGC as when using a dataset with 10 pulses m^−2^ in these fast-growing plantations. Relative root mean square errors (RMSEs) for the RF5 and RF10 were 6.14 and 6.01%, respectively. Equivalence tests showed that the predicted AGC from the training and validation models were equivalent to the observed AGC measurements. The grid cell sizes for mapping ranging from 5 to 20 also did not significantly affect the prediction accuracy of AGC at stand level in this system.

**Conclusion:**

LiDAR measurements can be used to predict and map AGC across variable-age Eucalyptus plantations with adequate levels of precision and accuracy using 5 pulses m^−2^ and a grid cell size of 5 m. The promising results for AGC modeling in this study will allow for greater confidence in comparing AGC estimates with varying LiDAR sampling densities for Eucalyptus plantations and assist in decision making towards more cost effective and efficient forest inventory.

## Background

Atmospheric carbon dioxide concentration [CO_2_] has increased by 40% since pre-industrial times, contributing greatly to climate change [1]. Managing the exchange of CO_2_ and other greenhouse gases between the biosphere and the atmosphere is an important strategy for mitigating climate change [2]. Forest ecosystems play a key role in the global carbon cycle [3–6], since carbon is exchanged naturally between forests and the atmosphere through photosynthesis, respiration, decomposition and combustion [7]. Forest management can therefore alter the amount and magnitude of CO_2_ exchange between forests and the atmosphere, thus serving as an effective and economical means to help mitigate climate change [8].

Forest plantations cover approximately 1% of the tropics (40–50 million ha) and have great capacity to store carbon [9], both during growth and in the form of durable forest products after harvest. Forest plantations play a role in terms of carbon in reducing further degradation and deforestation of natural forests, as well as providing alternative to the fossil fuels. *Eucalyptus* spp. are fast-growing with desirable wood qualities and are, therefore, a preferred species in plantations; they are widely grown in the tropics and subtropics, especially in Brazil, India, and China [10]. At present, Eucalyptus is grown on more than 20 million ha of plantation land around the world [11]. Eucalyptus is the dominant hardwood plantation species in Brazil, where it has been planted on more than 3.1 million hectares, accounting for approximately 57% of the country’s total reforested area [12]. Most Eucalyptus plantations are managed in short rotations (6–8 years) and the mean annual increment is approximately 40 m^3^ ha^−1^ year^−1^ roundwood, ranging from 25 to 60 m^3^ ha^−1^ year^−1^ depending on the level of environmental stress [13]. Quantifying the substantial roles of fast-growing Eucalyptus plantation on AGC stores, as sources of carbon emissions and as carbon sinks, has become key to understanding the global carbon cycle [6, 14].

Forest inventory in Eucalyptus plantations is usually conducted annually to monitor forest growth [13]. The aboveground carbon (AGC) production of *Eucalyptus* spp. is extremely high compared to natural forests especially when trees are grown for timber production and therefore contributes strongly to the reduction of atmospheric CO_2_ [9, 14, 15]. Remote sensing of forest AGC has received increased attention during the last decade due to its relevance to global carbon cycle modelling and to international programs aimed at reducing greenhouse gas emissions [15, 16]. LiDAR (Light Detection and Ranging) is a powerful remote sensing technology for predicting forest attributes [17], since it enables precise mapping of the landscape distribution of forest attributes at high spatial resolution and in a relatively short time compared to conventional methods [15]. In particular, many studies showed good relationships between parameters derived from airborne LiDAR data and forest measures such as canopy height [18–20], basal area [21, 22], stem volume [23–26] and aboveground carbon [7, 15, 27–31].

While airborne LiDAR is increasingly used to map forest attributes at the landscape level, multitemporal LiDAR data acquisition over large areas is expensive [32]. Many factors influence the cost of LiDAR data, including normal cost variables, such as project size, location, and deliverables, as well as market variables, such as competition amongst LiDAR vendors. One of the most important variables affecting the cost of acquisition of LiDAR data is pulse density [33, 34]. Pulse density is defined by the number of pulses sent by the sensor per m^2^ (pulses m^−2^), and as pulse density increases the acquisition cost increases as well [32].

There has been a wide interest in understanding how reducing pulse density affects the accuracy of inventory information derived from LiDAR data [34–37]. For instance, a study conducted in a Douglas-fir plantation in South Island, New Zealand, found that the precision of models to predict forest attributes, such as mean height, volume and mean diameter at breast height (DBH), remained stable until densities of 10 pulses m^−2^ were culled to 2–3 pulses m^−2^ [38]. Moreover, they also found that for the scenario where the DTM created from high pulse density was used to height normalize a downscaled point clouds for corresponding LIDAR-derived metrics, little change in the precision of regression models was found until pulse densities of 0.2–0.04 pulses m^−2^ were reached [38]. In mixed conifer forest in Washington state, USA, model precision was more affected by sample plot size than pulse density [39]. In mixed conifer-hardwood in Canada, pulse density could be reduced from 3.2 to 0.5 pulses m^−2^ with little effect on the quality of inventory results [40].

Besides the effect of pulse density on forest attribute prediction, there is also interest in understanding how cell size combined with pulse density affects the prediction of AGC at the stand level. Most LiDAR studies have been mapping forest attributes at stand level with grid cell size similar to the size of the sample plots used for calibrating the models [19, 22, 37], which is the general recommendation [42]. However, the shapes of the inventory plots are usually different from the cell shapes utilized in the prediction maps [43] that are normally square. It seems that at high pulse density, it would be advantageous to map forest attributes with higher spatial precision; i.e., at a finer grid resolution than the plot size, especially in Eucalyptus plantation, where the trees are planted in a grid and in most cases are hybrid clones. However, it is not clear how different grid cell sizes could influence stand level forest attributes estimates due to the degree of variability within stands and how LiDAR predictions are derived within a cell [43]. For instance, a study evaluating the influence of six prediction cell sizes (2, 4, 10, 25, 50, and 100 m squares) and two prediction methods (parametric vs. non-parametric) on LiDAR-derived stand-level estimates of total volume found not trend of smaller cell sizes producing lower values of volume, while larger cell sizes tended to give higher estimates [43].

Understanding the combined influence of pulse density and grid cell sizes on AGC prediction and mapping allows us to understand how well AGC estimates can be compared over time, as LiDAR technology and flight characteristics vary. Although the effects of LiDAR pulse density on the prediction accuracy of forest attributes has been widely investigated in boreal and temperate forests [33–36, 38–40], no studies have been conducted in fast-growing plantations with the goal of assessing the combined effect of pulse density and grid cell size on predicting and mapping aboveground carbon. Therefore, the aim of this study was first to predict AGC in eight fast-growing Eucalyptus plantations in southeast Brazil using metrics derived from LiDAR with pulse densities of 5 and 10 pulses m^−2^; and second, to assess the combined effect of these two pulse densities and grid cell sizes on AGC predictions at plot and stand levels. We hypothesized that due to the high homogeneity of this type of plantation, LiDAR data with a density of 5 pulses m^−2^ would provide AGC prediction accuracies similar to those from LiDAR data with a density of 10 pulses m^−2^, at grid cell sizes ranging from 5 to 20 m.

## Methods

### Study area description

**Fig. 1:**
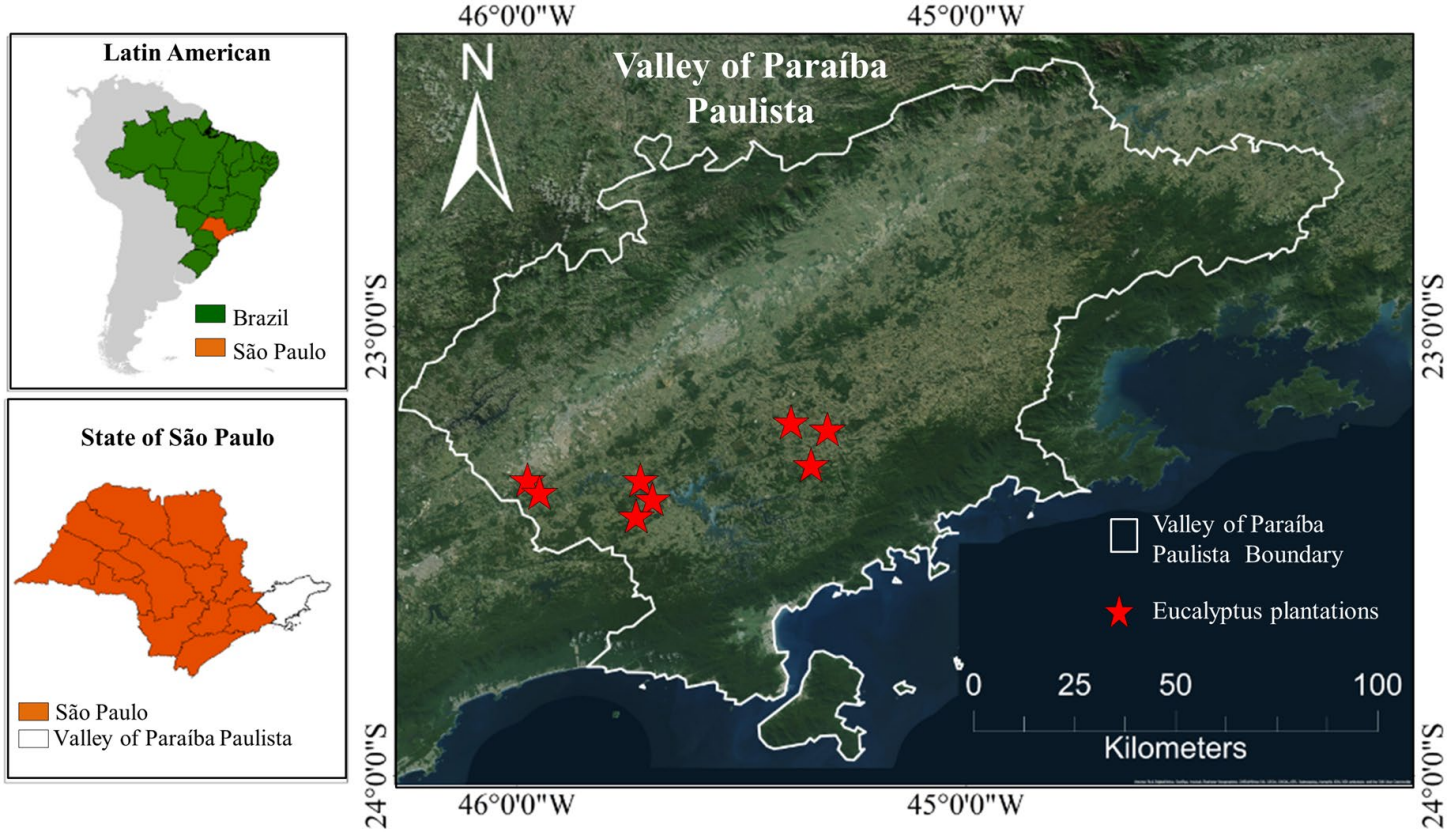
Location of the study area in the State of São Paulo, Brazil. The *stars* indicate the location of the Eucalyptus plantations

The study area consisted of eight farms located within the Paraíba Valley in the state of São Paulo, Brazil (Fig. 1). The climate of the region is characterized as humid subtropical, with dry winters and hot summers [41]. Mean annual precipitation is ~1200 mm; mean temperature ranges from a minimum of 17.1 °C in the coldest month (July) to a maximum of 23.9 °C in the hottest month (February). The topography in the selected plantations is complex with high relief, ranging from 578 to 1310 m in elevation. The plantations contain hybrid clones of two Eucalyptus species, *Eucalyptus grandis* W. Hill ex Maid and *Eucalyptus urophylla* S.T. Blake. The plantations are managed by Fibria Celulose S/A, a pulp company. Stand age across the farms was variable and ranged from 3 to 8 years. All the trees were planted in 3 m between rows and 2 m tree within a row (1667 trees per ha).

### Field data collection

A total of 136 circular plots of 400 m^2^ each were established across the eight farms (Site ID, Table 1) for stand measurement during the months of January and March of 2012. All plots were georeferenced with a geodetic GPS with differential correction capability (Trimble Pro-XR). Sample plot centers were geolocated with a horizontal error of up to 10 cm. In each plot, all trees were measured for diameter at breast height (DBH; cm) and a random subsample (15%) of trees for maximum height (Ht; m). For trees in the plot that were not directly measured for Ht, the inventory team of Fibria Celulose S/A company predicted heights from hypsometric models, which use DBH as the predictor of Ht. For mapping validation purpose, trees were sub-sampled in square sub-plots of 25 m^2^ (5 × 5 m), 100 m^2^ (10 × 10 m) and 225 m^2^ (15 × 15 m) located at the center of each plot. The total aboveground carbon—AGC (Mg.tree^−1^) for the plots and sub-plots was obtained through the allometric model according to [15], employing as independent variables the logarithm of DBH and the Ht, and as dependent variables the AGC, as shown in the equation below:

$${ \ln }\left( {\text{AGC}} \right) = - 2. 8 7 + 1. 9 5 { } \times { \ln }\left( {\text{DBH}} \right) + 0. 4 4\times { \ln }\left( {\text{Ht}} \right)$$

where: DBH is tree diameter over bark at breast height (1.37 m) in cm; Ht is tree height (m). The AGC model has adjusted coefficients of determination (Adj.R^2^) of 0.97, absolute and relative root mean squared errors (RMSE) of 4.57 kg tree^−1^ and 12.38%, respectively. The ln (AGC) was back transformed to natural scale, and multiplied by a correction factor of 1.03 [exp (0.5 × MSE)] to remove the bias added by the log transformation. The summed AGC stock of all trees within the plots and sub-plots was then divided by the plot and sub-plot area to calculate the AGC at plot-level in Mg.ha^−1^. The summary statistics of AGC stocks (Mg.ha^−1^) measured at plot-level in the farms evaluated are presented in Table 1.

**Table 1:** Descriptive values of the biometric parameters of the network of plots inventoried in the study area (January of 2012)

Sites ID	Area (ha)	DBH (cm)		Ht (m)		AGC (Mg.ha^−1^)		Age (years)	N plots
		Mean	SD	Mean	SD	Mean	SD		
F987	39.53	8.82	1.57	11.65	2.55	15.76	3.75	2.3	14
F986	94.16	12.73	0.76	18.60	1.45	45.45	6.19	3.3	21
F849	138.96	14.14	0.60	22.16	1.54	62.71	6.69	4.7	26
F950	86.72	13.73	0.80	21.46	1.39	62.97	9.81	5.5	17
F184	58.34	14.57	0.70	23.74	1.19	64.59	6.81	5.9	14
F166	84.35	14.59	0.95	24.17	1.38	69.76	9.77	6.1	17
F948	79.33	13.70	1.20	23.29	1.68	60.42	13.12	6.8	12
F634	84.80	15.26	1.20	25.24	2.35	73.75	9.87	8.0	15

*SD* standard deviation

### LiDAR data acquisition and data processing

LiDAR data were obtained with a Riegl LMS-Q680I sensor mounted on a Piper Seneca II aircraft during the months of January and March of 2012. Data were acquired subsequently with pulse densities of 5 and 10 pulses m^−2^; the characteristics and precision of the LiDAR data are listed in Table 2. LiDAR data processing consisted of several steps that ingested the LiDAR point cloud data and provided two major outputs: the digital terrain model (DTM) and the LiDAR-derived canopy structure metrics.

**Table 2:** LiDAR flight characteristics

Parameter	Value
Average flight height	422.94 m
Pulse density	5 and 10 pulses m^−2^
Pulse frequency	400 kHz
Scan angle	±45º
Laser wavelength	1055 nm
Average aircraft speed	57 m/s (205.20 km/h)
Horizontal precision	0.1–0.15 m (1.0 sigma)

LiDAR data processing for both pulse densities occurred with the following sequence of steps using FUSION/LDV toolkit software [44]. (I) Ground returns were classified using a filtering algorithm adapted from [45] based on linear prediction [46]. (II) Digital Terrain Models (DTMs) of 1 m spatial resolution were then developed using the classified ground returns. (III) Normalized LiDAR point clouds were obtained by subtracting the DTM elevation from each LiDAR return. (IV) Normalized point clouds were subset within each of the 136 sample plots, and then structure metrics were computed for each plot using only all returns above 2 m to remove the returns not belonging to tree crowns (e.g., hits on near-ground shrubs, forbs, grasses, etc.). (V) To evaluate the effect of grid cell size on the AGC prediction at stand level, we computed LiDAR metrics at grid cell sizes of 5, 10, 15, and 20 m. From the point cloud, it is possible to generate many LiDAR metrics, however, we generated only those metrics that have been frequently used as candidate predictors for forest attribute modeling in other studies [15, 18–22, 29, 47]. Therefore, a total of 27 LiDAR metrics calculated from all returns were considered for AGC modelling at the plot and stand levels (Table 3).

**Table 3:** LiDAR-derived canopy height metrics considered as candidate variables for predictive AGC models [43]

Metric	Description
HMIN	Minimum canopy height
HMAX	Maximum canopy height
HMEAN	Mean canopy height
HMAD	Median canopy height
HSD	Standard deviation of canopy height
HSKEW	Skewness of canopy height
HKURT	Kurtosis of canopy height
HVAR	Variance of canopy height
HCV	Coefficient of variation canopy height
HMODE	Mode canopy height
H01TH	1st percentile of canopy height
H05TH	5th percentile of canopy height
H10TH	10th percentile of canopy height
H20TH	20th percentile of canopy height
H25TH	25th percentile of canopy height
H30TH	30th percentile of canopy height
H40TH	40th percentile of canopy height
H50TH	50th percentile of canopy height
H60TH	60th percentile of canopy height
H70TH	70th percentile of canopy height
H75TH	75th percentile of canopy height
H80TH	80th percentile of canopy height
H90TH	90th percentile of canopy height
H95TH	95th percentile of canopy height
H99TH	99th percentile of canopy height
CR	Canopy relief ratio [(HMEAN-HMIN)/(HMAX-HMIN)]
COV	Canopy cover (percentage of first return above 1.30 m)

### Random forest modeling

We used the random forest package [48, 49] in R [50] to create random forest (RF) models for predicting AGC at the plot and stand levels from the LiDAR dataset of 5 and 10 pulses m^−2^ (RF5 and RF10). In the analysis, we defined the number of classification trees (ntree) as 1000, and for the number of variables randomly (mtry) sampled as candidates at each split, we used the default value, which for regression is defined as p/3, where p is the number of covariates. For the remaining parameters, we used the default values, which are all defined in [50] as well.

The selection of the best LiDAR metrics to be included in the models was performed in two steps. First, even though highly correlated variables won’t cause multi-collinearity issues in RF, Pearson’s correlation (r) was used to identify highly correlated predictor variables (r > 0.85) as presented in [29] and [15, 47]. If a given group (2 or more) of LiDAR metrics were highly correlated, we retained only one metric by excluding the others that were most highly correlated with the retained metrics. Second, we identified the most important metrics based on the Model Improvement Ratio (MIR), a standardized measure of variable importance [51, 52]. MIR scores are derived by dividing raw variable important scores (output from RF) by the maximum variable importance score, so that MIR values range from 0 to 1. MIR scores allow for variable importance comparisons among different RF models. For each of the two AGC models, we ran 1000 iterations of RF that included the <0.85 correlated preliminary set of LiDAR metrics to create distributions of MIR for each metric. Running 1000 iterations of RF produced consistent MIR distributions and avoided unnecessary processing time. To create parsimonious models, we reserved metrics for final RF models that were consistent and exhibited the highest mean MIR values.

The accuracy of estimates for each model was evaluated in terms of percentage of variation explained (Pseudo-R^2^) in the RF models, Root Mean Square Error (RMSE), and Bias (both absolute and relative) computed by the linear relationship between observed and predicted AGC:

1 $$Pseudo - R^{2} = \left[ {1 - \frac{{\mathop \sum \nolimits_{i = 1}^{n} \left( {y_{i} - \hat{y}_{i} } \right)^{2} }}{{\mathop \sum \nolimits_{i = 1}^{n} \left( {\frac{y}{n } - y_{i} } \right)^{2} }}} \right]$$

2 $$RMSE = \sqrt {\frac{{\mathop \sum \nolimits_{i = 1}^{n} \left( {y_{i} - \hat{y}_{i} } \right)^{2} }}{n}}$$

3 $$Bias = \frac{1}{n}\mathop \sum \limits_{i = 1}^{n} \left( {y_{i} - \hat{y}_{i} } \right)$$

where n is the number of plots, y_i_ is the observed value for plot i, and $\hat{y}_{i}$ is the predicted value for plot i. Moreover, relative RMSE and biases were calculated by dividing the absolute values (Eqs. 1, 2) by the mean of the observed AGC. We defined acceptable model precision and accuracy as a relative RMSE and Bias of ≤15% to have a model precision and accuracy higher than or equal to the conventional forest inventory standard in fast-growing Eucalyptus forest plantations in Brazil [14, 47].

The performance of the RF models to predict AGC was also evaluated by means of a leave-one-out cross-validation (LOOCV) strategy [e.g. 22, 49]. Statistical equivalence tests were employed [53, 54] to assess whether the AGC predictions were statistically similar at (i.e., equivalent; *p* value >0.05) to the field-based AGC stocks. Following [55], we employed a regression-based equivalence test for intercept equality to 0 (i.e., the mean of predicted AGC is equal to the mean of the field-based AGC) and slope equality to 1 (i.e., if the pairwise, predicted and observed AGC are equal, the regression will have a slope of 1). A description of equivalence tests can be also found in the ‘equivalence’ package in R [56], and examples of equivalence plots in LiDAR studies can be found in [15, 29, 45, 53, 55]. We also computed RMSE, Bias and the adjusted Coefficient of determination (Adj.R^2^) from the observed and predicted AGC from LOOCV. Finally, the effects of pulse density of the AGC predictions at the plot-level were investigated by the comparison of RMSE and Bias statistics across pulse densities.

### Assessing effects of cell size on the AGC prediction and mapping at the stand level

**Fig. 2:**
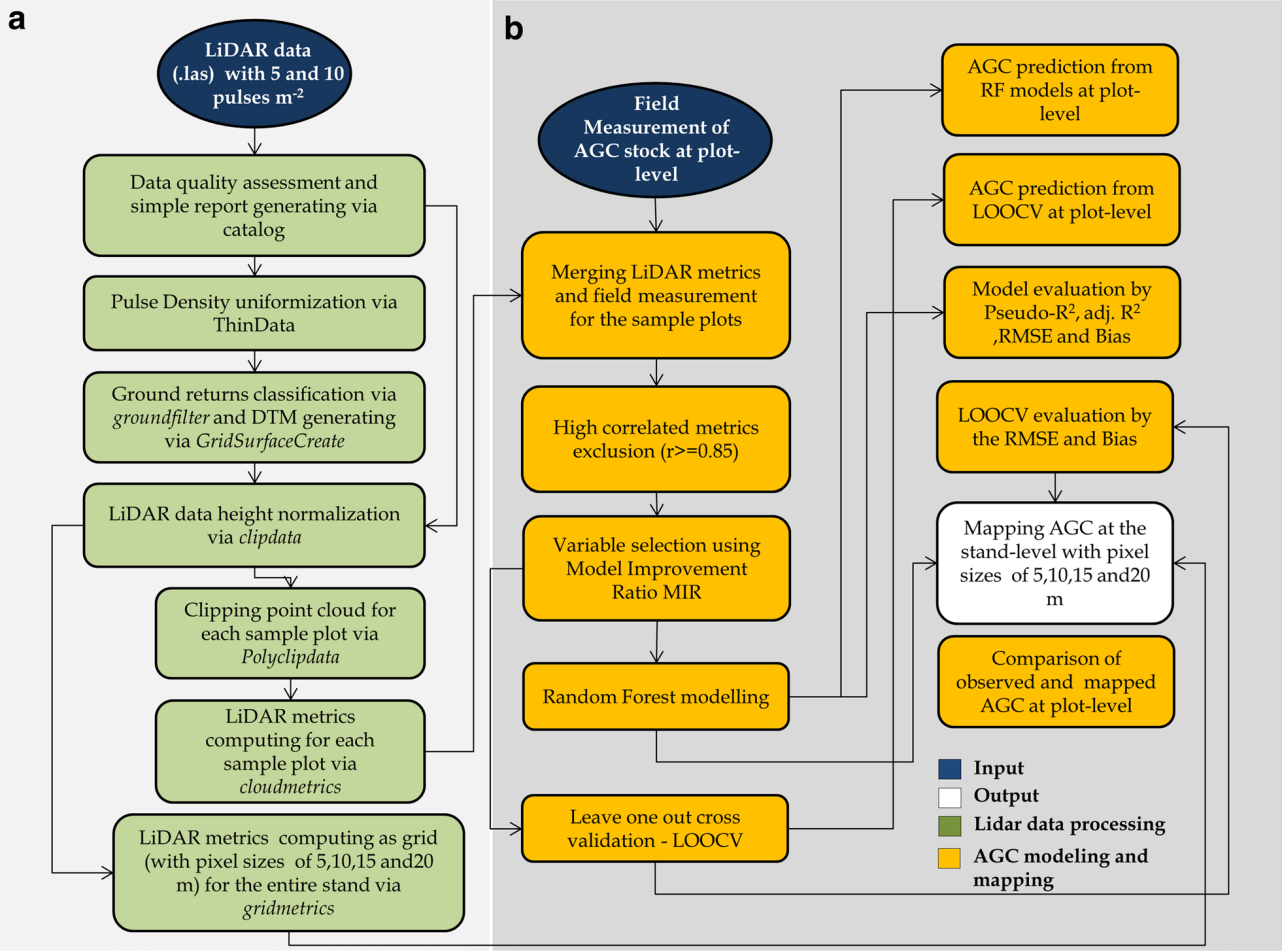
Procedure for predicting and mapping AGC from LiDAR and inventory plot data in Eucalyptus fast-growing plantations. Pearson’s correlation (r); Model Improvement Ratio (MIR); Leave-one-out cross-validation (LOOCV); Adjusted coefficient of determination (adj.R^2^); Percentage of variation explained (Pseudo-R^2^); Root mean squared error (RMSE). **a** The light gray panel corresponds to the LiDAR data processing step and **b** the gray panel correspond to the AGC modeling step

As the study area is characterized by fast-growing plantations, with tree hybrid clones equally spaced in the ground, we assumed that AGC maps could be generated with high spatial resolution, even though the model is based on a sample unit of 400 m^2^. Therefore, we applied the predictive model across the landscape to map AGC at spatial resolutions of 5, 10, 15, and 20 m, the last of which equates to the sample plot size of 400 m^2^. To evaluate the effect of grid cell size on the AGC map prediction, we extracted the AGC for each study site, and boxplots were generated to compare variability of AGC at the stand level containing various grid cell sizes. Moreover, from the maps we extracted predicted AGC within the plot and sub-plot boundaries to validate the maps by the comparison with reference AGC through an equivalence test [53–57]. An overview of the methodology is outlined in Fig. 2.

## Results

### Canopy profile of Eucalyptus plantation across age and LiDAR pulse density

**Fig. 3:**
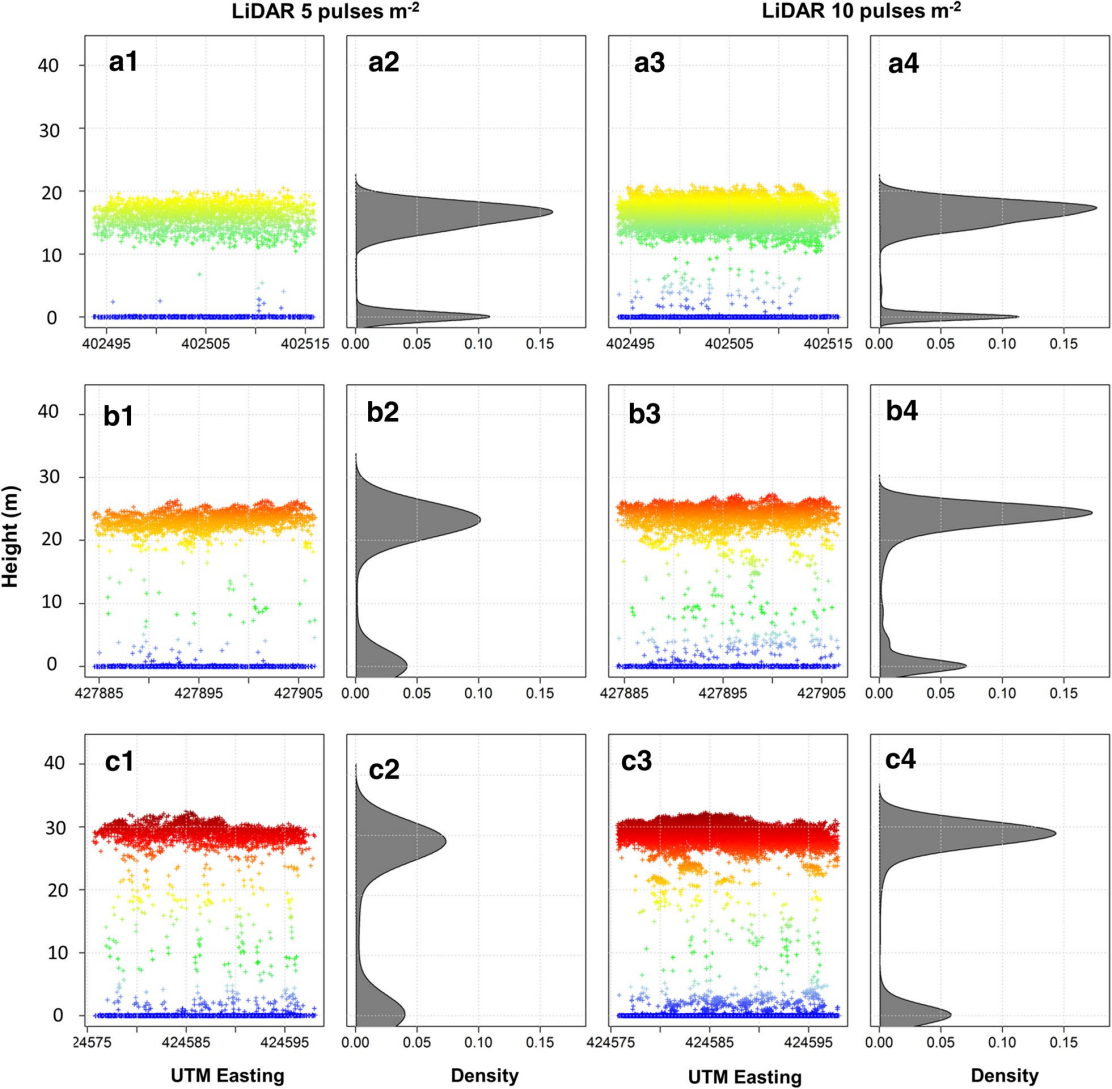
LiDAR profiles of selected sample plots (400 m^2^) of Eucalyptus representative of early (i.e. 3.3 years) (**a**), intermediate (i.e. 5.5 years) (**b**) and advanced stages of development (i.e. 7.9 years) (**c**). (*1*) and (*3*) UTM easting profiles, (*2*) and (*4*) density plots. The *colors* represent height

The canopy profiles of Eucalyptus plantations across age and pulse density are represented in Fig. 3. LiDAR-derived height increased from early (Fig. 3a) to intermediate (Fig. 3b) and advanced ages (Fig. 3c). The variation in height was affected more by stand age than by pulse density. From a probabilistic perspective, the canopy height distribution remained the same using datasets with 5 and 10 pulses m^−2^. The majority of LiDAR returns came from the upper canopy and the ground. However, as the stands approached harvest age (e.g. ~7–8 years), the number of LiDAR returns in the 0–5 m strata increased, indicating that the understory plant community increased in cover and height as the stand ages.

### LiDAR metrics selection

Pearson’s correlation test (r) applied to the 27 candidate LiDAR metrics determined that 18 metrics were highly correlated (r > 0.85) for models using the 5 and 10 pulses m^−2^. For each dataset, we excluded all but one of the highly correlated metrics (H99TH), and the remaining not highly correlated metrics were used to build prospective models after MIR analysis. The not highly correlated LiDAR metrics derived from the 5 and 10 pulses m^−2^ dataset were HVAR, HCV, HSKE, HKUR, H01TH, H05TH, H10TH, H99TH and COV. In both correlation matrices for the LiDAR metrics derived from the 5 and 10 pulses m^−2^, there were positively correlated metrics, such as H99TH and HVAR (r = 0.65; r = 0.66; p value < 0.001), and negatively correlated metrics, such as HCV and COV (r = −0.34; r = −0.30; p value <0.001), as shown in Table 4.

**Table 4:** Pearson’s correlation among the derived metrics from LiDAR pulse density of 5 (white) and 10 (gray) pulses m^−2^

r	HVAR	HCV	HKUR	H01TH	H05TH	H10TH	H99TH	COV
HVAR	–	*0.84* ^***^	−*0.50* ^***^	−*0.44* ^***^	−*0.50* ^***^	−*0.30* ^***^	*0.66* ^***^	−*0.21* ^*^
HCV	0.86^***^	–	−*0.73* ^***^	−*0.58* ^***^	−*0.79* ^***^	−*0.66* ^***^	*0.25* ^**^	−*0.30* ^***^
HKUR	−0.53^***^	−0.73^***^	–	*0.50* ^***^	*0.83* ^***^	*0.70* ^***^	*0.06*	*0.10*
H01TH	−0.48^***^	−0.63^***^	0.51^***^	–	*0.53* ^***^	*0.26* ^***^	−*0.13*	−*0.29* ^***^
H05TH	−0.57^***^	−0.80^***^	0.85^***^	0.57^***^	–	*0.75* ^***^	*0.17* ^*^	*0.07* ^**^
H10TH	−0.37^***^	−0.67^***^	0.71^***^	0.26^**^	0.74^***^	–	*0.44* ^***^	*0.40* ^***^
H99TH	0.65^***^	0.29^***^	0.02	−0.18^*^	0.10	0.39^***^	–	*0.49* ^***^
COV	−0.30^***^	−0.34^***^	0.12	0.08	0.25^**^	0.18^*^	0.45^***^	–

The italic values indicates the values of r for metrics derived from 10 pulse m^−2^

*** p value <0.001; ** p value <0.01; * p value <0.05; p value <0.1

The remaining, not highly correlated LiDAR-derived metrics from 5 and 10 pulses m^−2^ were correlated against each other and metrics such as H99TH and HVAR where highly stable across pulse density, while COV and H01TH showed to be less stable. Pearson’s correlation between not highly correlated LiDAR-derived metrics from 5 and 10 pulses m^−2^ are shown in the Table 5.

**Table 5:** Pearson’s correlation between selected metrics from LiDAR pulse densities of 5 and 10 pulses m^−2^

r	LiDAR-derived metrics (10 pulses m^−2^)							
	HVAR	HCV	HKUR	H01TH	H05TH	H10TH	H99TH	COV
LiDAR-derived metrics (5 pulses m^−2^)								
HVAR	0.93^***^							
HCV	0.76^***^	0.87^***^						
HKUR	−0.38^***^	−0.55^***^	0.69^***^					
H01TH	−0.38^***^	−0.46^***^	0.40^***^	0.60^***^				
H05TH	−0.37^***^	−0.60^***^	0.60^***^	0.36^***^	0.68^***^			
H10TH	−0.25^**^	−0.55^***^	0.61^***^	0.17	0.61^***^	0.90^***^		
H99TH	0.64^***^	0.28^***^	0.03	−0.17^*^	0.11	0.40^***^	1.00^***^	
COV	0.14	−0.09^***^	0.13	−0.19^*^	0.18^*^	0.45^***^	0.50^***^	0.13

*** p value <0.001; ** p value <0.01; * p value <0.05; p value <0.1

The most important LiDAR metrics for predicting AGC, both from the 5 and 10 pulses m^−2^ datasets, were H99TH and HVAR. In addition to these two metrics, the H10TH and HSKE were also the most consistently important metrics with higher MIR (Table 6). The variation in metrics selection was less affected by pulse density, and the LiDAR metrics selected according to MIR were the same for both pulse densities.

**Table 6:** Mean of model improvement ratio (MIR) among derived metrics from LiDAR pulse density of 5 and 10 pulses m^−2^ (N = 1000)

Pulse density (pulse m^−2^)	LiDAR-derived metrics							
	**H01TH**	H05TH	H10TH	H99TH	HVAR	HCV	HKUR	COV
5	0.04	0.13	*0.47*	*1.00*	*0.68*	0.21	0.17	0.02
10	0.03	0.11	*0.33*	*1.00*	*0.52*	0.13	0.07	0.07

MIR > 0.3 are highlighted in italics

### RF model fit and cross validation

Both RF models predicting AGC at plot level explained 83% of the variation in AGC. Greater pulse density did not improve model fit, and both RF5 and RF10 models resulted in a very low RMSE and Bias, indicating high model prediction accuracy. In addition to the RMSE and Bias, r and adj.R^2^ did not significantly differ between the RF5 and RF10 models (Table 7).

**Table 7:** Random Forest models fitted for predicting AGC from LiDAR pulse densities of 5 and 10 pulses m^−2^

Pulse density	LiDAR derived metrics	Pseudo-R^2^	RMSE		Bias	
			Mg ha^−1^	%	Mg ha^−1^	%
5	H99TH + HVAR + H10TH	81.79	3.52	6.14	−0.03	−0.06
10		82.17	3.44	6.01	−0.01	−0.01

Pearson’s correlation coefficient (r); root mean square error (RMSE)

**Fig. 4:**
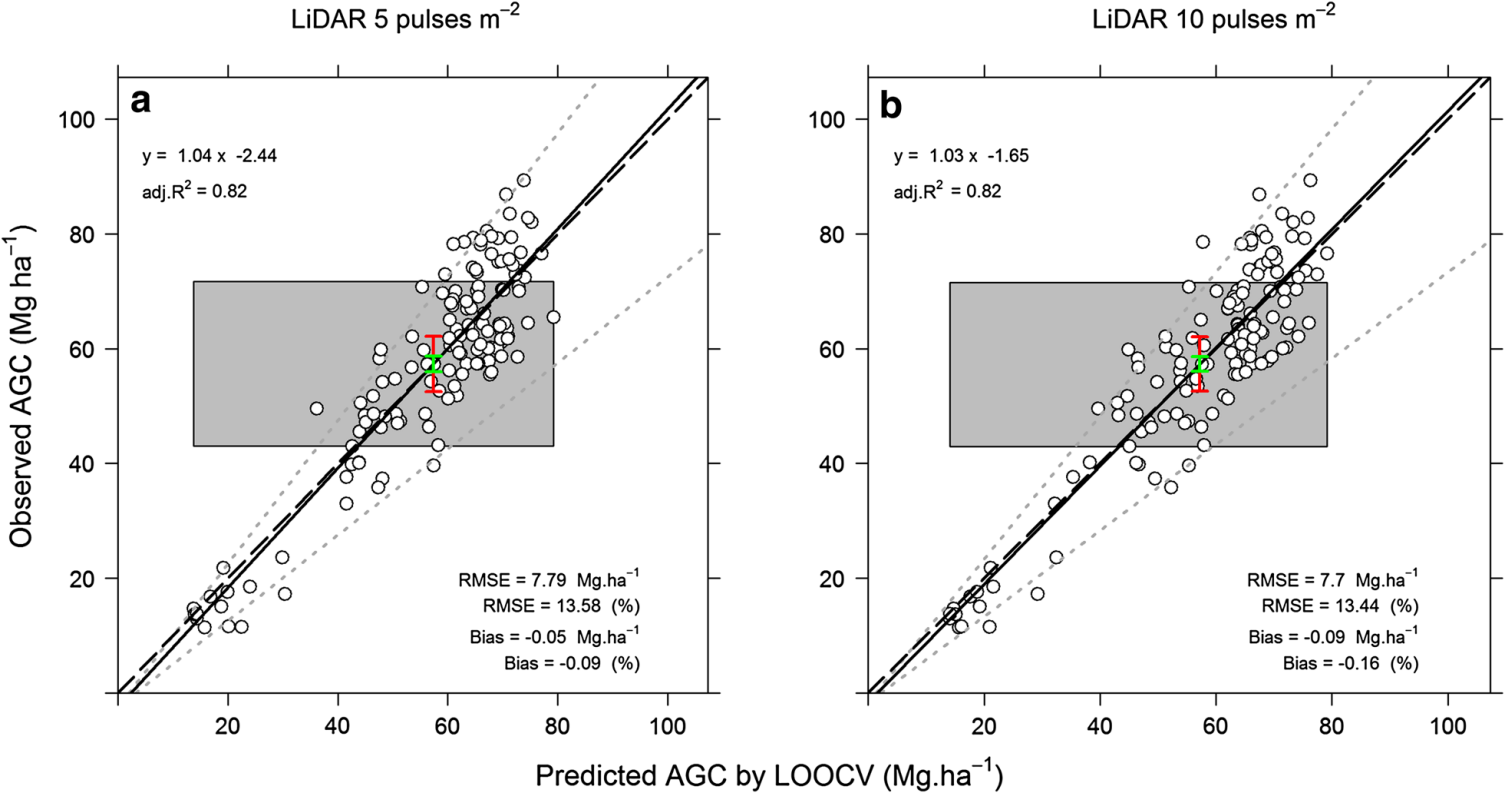
Equivalence plots of observed vs. predicted AGC by the LOOCV5 and LOOCV10 (**a**, **b**); (n = 136). The equivalence plot design presented herein is an adaptation of the original equivalence plots presented by [52]. The *grey polygon* represents the ±25% region of equivalence for the intercept, and the *red vertical bar* represents a 95% of confidence interval for the intercept. The predicted AGC from the LOOCV5 and LOOCV10 models are equivalents to the reference for the intercept if the *red bar* is completely within the *grey polygon*. If the *grey polygon* is lower than the *red vertical bar*, the predicted AGC is biased low; and if it is higher than the *red vertical bar*, the predicted AGC is biased high. Moreover, the *grey dashed line* represents the ±25% region of equivalence for the slope, and if the *green vertical bar* is contained completely within the *grey dashed line*, the pairwise measurements are equal. *Red and green bars* are wider than the region outlined by the *grey dashed lines* indicates highly variability predictions. The *white dots* are the pairwise measurements, and the *solid line* is a best-fit linear model for the pairwise measurements. The *light grey dashed line* represented the relationship 1:1

The LOOCV analysis indicated that the RF5 and RF10 models were stable resulting RMSE of 13.61 and 13.38% and Bias of −0.09 and −0.27% for the models derived from the 5 and 10 pulses m^−2^ datasets, respectively. Moreover, the equivalence plots of observed versus predicted AGC both from the two models showed that predicted and observed AGC were equivalent (p values >0.05 for intercepts and p values <0.05 for slopes (Fig. 4a, b).

### AGC predictions at plot-level

**Fig. 5:**
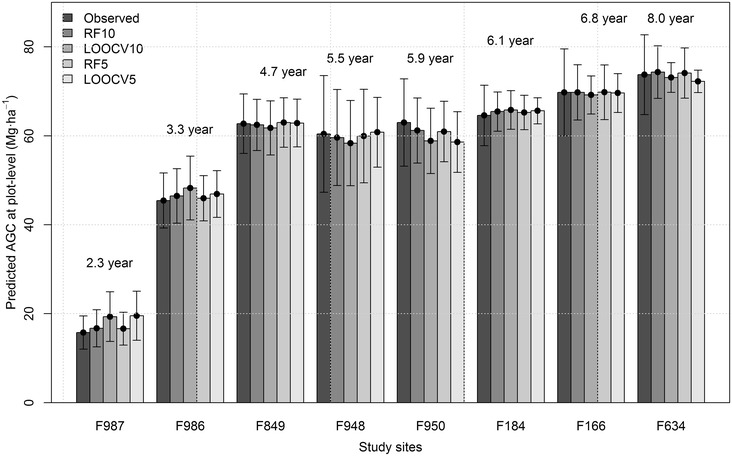
Observed versus predicted AGC by the RFs and LOOCVs for each plantation in the study area. *Black dots* represent averages and *vertical bars* represent standard deviations of AGC

There was no difference in the AGC prediction at plot level using LiDAR data of 5 or 10 pulses m^−2^. The RF model AGC predictions for the 136 sample plots ranged from 12.99 to 82.37 for RF5, and 12.75 to 82.83 Mg.ha^−1^ for RF10. In general, for both RF5 and RF10 the AGC values were slightly overestimated during early and advanced stand ages, and slightly underestimated at intermediate ages. The Eucalyptus plantations containing younger stands showed the lowest AGC values (i.g. IDs F987 and F986), and advanced age stands contained the highest AGC stocks (i.g. IDs F166 and F634) (Fig. 5).

### Effect of the pulse density and cell size on the AGC prediction and mapping at stand level

**Fig. 6:**
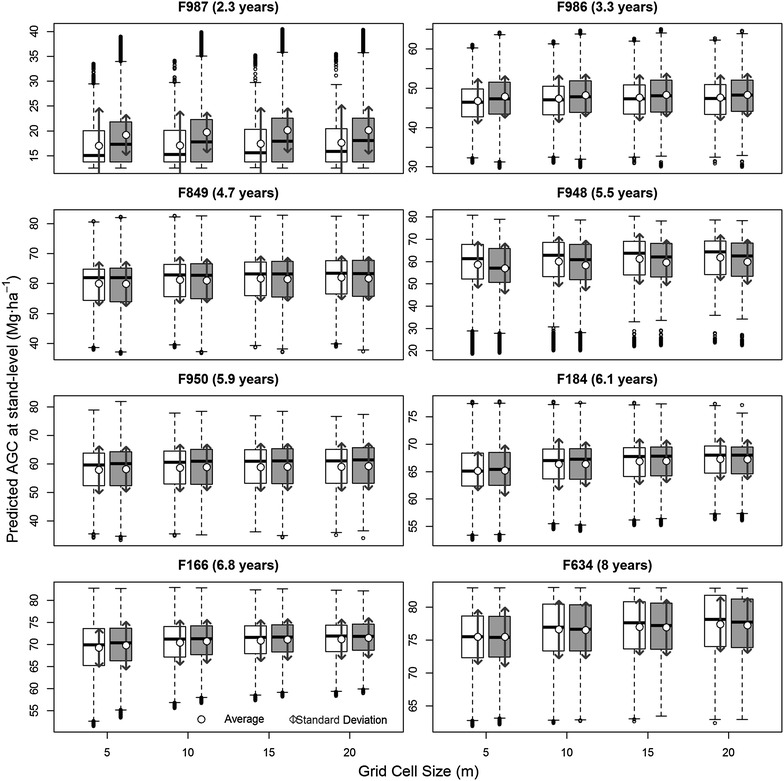
Predicted AGC at stand level. The *white and gray boxplots* represent the AGC predicted from 5 and 10 pulses m^−2^, respectively

The variation of the predicted AGC at stand level, and across cell size and pulse density is presented in Fig. 6. Grid cell sizes higher than 5 m did not significantly affect AGC prediction at stand level. Even though the variability of AGC slightly decreased from 5 to 20 m cell size, we observed that in most of the sites, the AGC variability was similar in the maps with spatial resolution coarser than 5 m.

**Fig. 7:**
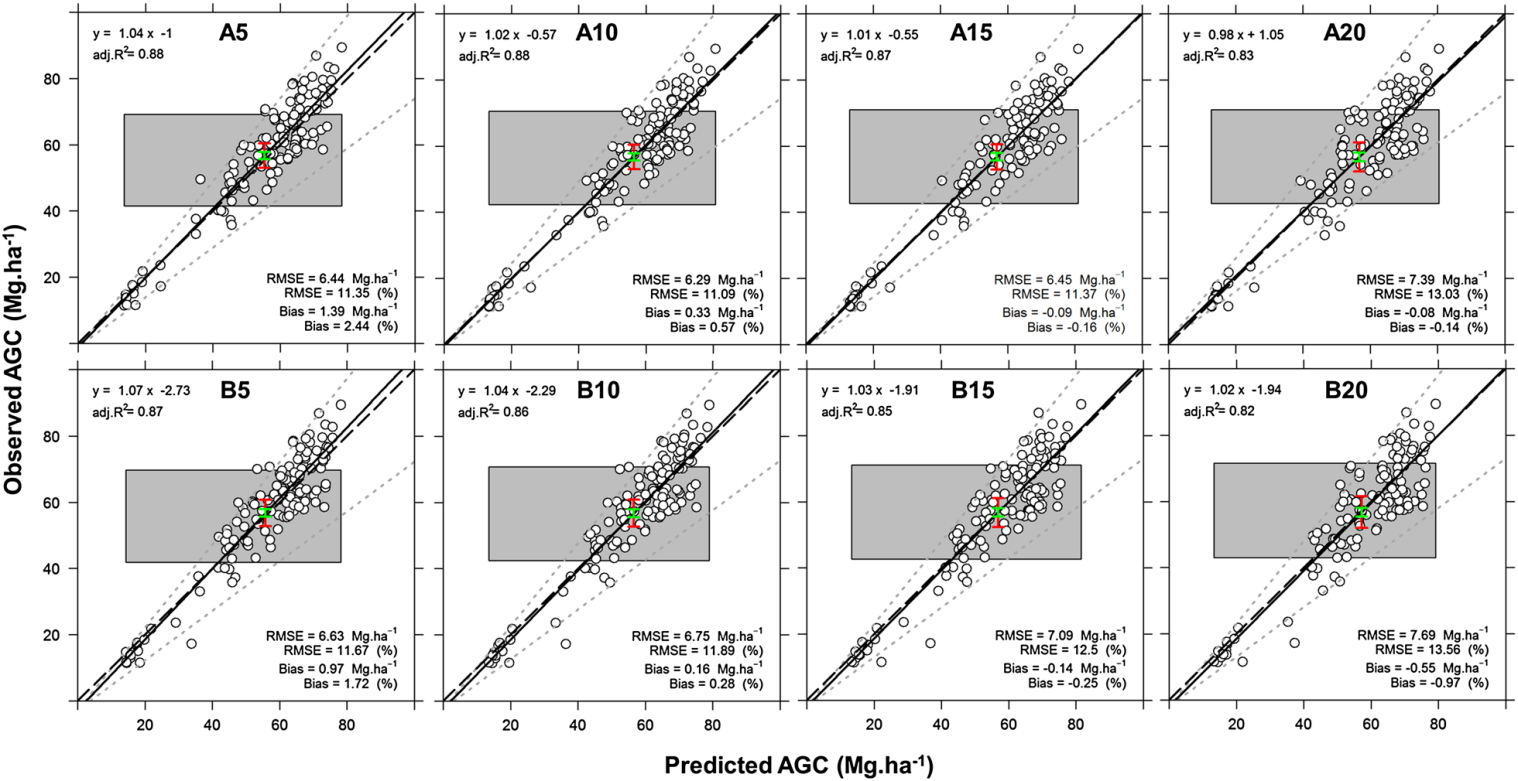
Equivalence plot of the observed and predicted AGC at stand level for the RF5 (**a**) and RF10 (**b**); (n = 136). The numbers *5*, *10*, *15*, and *20* m represent the grid cell sizes. The description of equivalence plot is presented in the caption of Fig. 4

For each sample plot we extracted the predicted AGC from the maps. Equivalence plots indicated that the observed and predicted AGC were equivalent for the maps with all grid cell sizes (p values >0.05 for intercepts and p values <0.05 for slopes). RMSEs and Bias ranged from 11.01 to 12.3% and −0.37 to 0.87%, and showed lower values in the maps with cell size of 5 m (Fig. 7). Although the observed and predicted AGC values were equivalents at spatial resolution of 5 m for both 5 and 10 pulses m^−2^, the maps are overestimating AGC in young stands (e.g. “F987”) and underestimating AGC in older stands (e.g. “F634”).

## Discussion

Forest managers are seeking new management strategies that integrate forest, industry and market goals that maximize financial return while ensuring sustainability in the forest production chain [14]. The carbon credit market has created optimistic prospects for expansion of the Brazilian forest sector. Although this market seems attractive, there are few studies available with carbon stock estimates for planted forests [15]. In this study, we evaluated the combined effect of pulse density and grid size on AGC prediction at plot and stand levels in a fast-growing Eucalyptus plantation. A significant amount of work has been done to evaluate the effect of LiDAR pulse density for forest inventory modeling. However, less attention has been given to the influence of the cell size used to generate stand-level maps of forest attributes [43].

Accurate and timely measurements of AGC are critical for understanding the structure and function of terrestrial ecosystems, as well as sustainable forest resources management and carbon accounting [29]. Eucalyptus plantations are a major carbon sink compared to others types of plantations, efficiently transferring atmospheric carbon into forest biomass and soil [10, 11]. The need to increase the accuracy and the spatial coverage of carbon accounting in these types of ecosystems has economic, politic and scientific rationales [31]. Airborne LiDAR can acquire tree-dimensional information of all eucalyptus trees at a high spatial resolution in a short time [15], and whether there is benefit to using LiDAR for forest inventory depends in large part on characteristics specific to each forest [35]. The results presented in this current study provide a validation of plot and stand-based measurements of AGC. LiDAR has been shown to be a powerful technology for AGC prediction in Eucalyptus plantations, and our results have demonstrated that highly accurate estimates of AGC can be achieved using LiDAR data.

Our results show that a LiDAR pulse density of 5 pulses m^−2^ provides similar AGC prediction accuracy to that using a dataset with 10 pulses m^−2^ in fast-growing eucalyptus plantations. Previous studies have demonstrated similar effect of pulse density in forest attribute estimation. For example, [21] found that pulse densities could be reduced from 1.13 to 0.25 pulses m^−2^ with little effect on the quality of the forest inventory results in stands dominated by Norway spruce (*Picea abies* L. Karst.) and Scots pine (*Pinus sylvestris* L.). [35], whose objective was quantifying the effects of LiDAR pulse density and sample size on forest attributes prediction from LiDAR-derived metrics, found that model precision was more affected by sample plot sizes than by pulse density in mixed conifer forest in Washington state, USA. In a remnant forests in the rapidly urbanizing region of Charlotte, North Carolina, USA, LiDAR point density was responsible for a biomass prediction difference of 11.5% when the pulse density was downscaled from 100 to 1% [58]. The statistical parameters adj.R^2^, RMSE and Bias presented in this study for the evaluation of AGC models are within the range reported by these other studies [33, 38, 47], though it is difficult to make a direct comparison due to differences in LiDAR sensors, pulse density testes, forest types and ground data collection.

LiDAR derived metrics, such as height percentiles, have been shown to be the most important LiDAR predictors of forest attributes in Eucalyptus plantations [15, 26, 47, 59]. In this study, the H99TH, H10TH, and HVAR were the most useful predictor variables for AGC prediction for the models derived from datasets of 5 and 10 pulses m^−2^. These selected LiDAR metrics succinctly described the 3-dimensional canopy structure by capturing the majority of variation contained in the point cloud. In particular, H99TH captured canopy top height, and HVAR captured the canopy height variation of Eucalyptus stands. Also, the H99TH and HVAR were the most stable LiDAR-derived metrics when compared across pulse densities, accounting for a positive and strong linear correlation of r = 1 (H99TH5 × H99TH10) and r = 0.93 (HVAR5 × HVAR10).

The most accurate method of estimating AGC in Eucalyptus plantation is to physically sample it in the field. In a conventional inventory, one sample plot (300–500 m^2^) per each 10 or 15 ha is normally measured with a goal of achieving a maximum acceptable RMSE of 10–15% [60]. However, this type of measurement over large areas is severely limited by cost and time. Approaches for deriving AGC information based on LiDAR data are of great utility and interest; in this study, we showed that airborne LiDAR with pulse densities of 5 pulses m^−2^ can be used to predict AGC over large areas with RMSE and Bias equal to or less than in a conventional inventory. In this study, we achieved the RMSE goal of <15% established in the Methods section.

AGC maps are useful for forest management because they show the distribution of the AGC stock across the whole area. In theory, as spatial resolution increases, the higher is the probability to detect finer details and variability in forest stands. In this study, we found that AGC maps with spatial resolution ranging from 5 to 20 m show the same variability of AGC at stand level, indicating that in a fast-growing Eucalyptus plantation it is possible to map AGC in high detail even though the model was fitted using sample plots with larger sizes than the grid cell size used for mapping. However, LiDAR metrics computed in a grid cell of 5 m captured finer details of forest structure variability then those computed in a grid cell of 20 m; therefore, maps overestimated AGC in young stands and underestimated AGC in older stands, because the models calibrated from tree data collected within plots of 400 m^2^ were not able to capture the forest variability at this fine resolution. The influence of the cell size used to generate forest attributes from LiDAR data, such as AGC, has not analyzed intensely at stand-level yet [43], and the range of examined cell sizes was limited [61, 62]. Whereas the differences among the cell sizes were almost negligible in [61], the effects of cell sizes on estimates was highlighted in [43], especially when comparing extreme cell sizes (2 and 100 m). These results concur with ours at resolutions between 5 and 20 m.

**Fig. 8:**
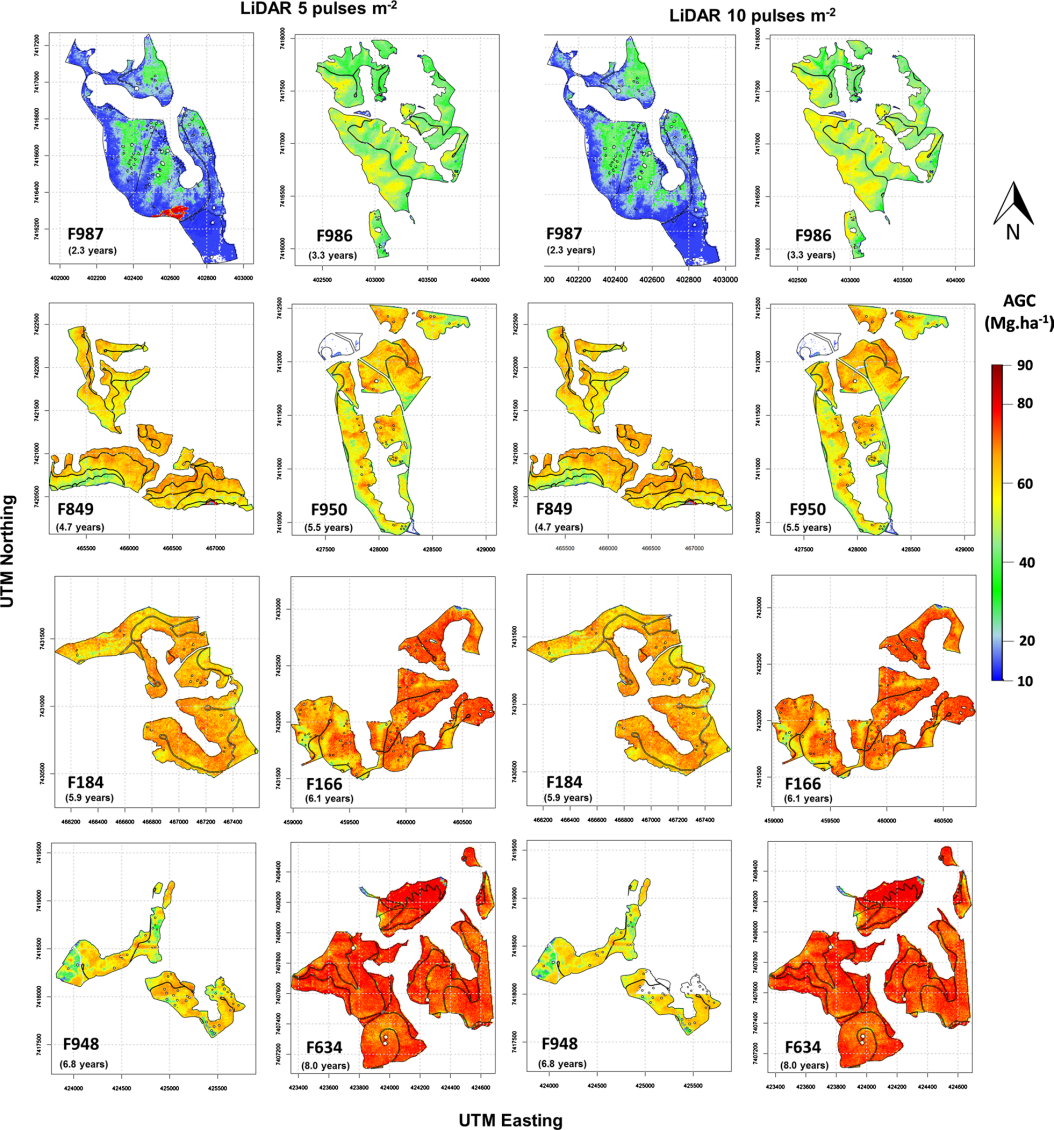
Predicted AGC of Eucalyptus at stand level with grid cell size of 5 m and pulse densities of 5 and 10 pulses m^−2^ (see Table 1 for the sites codes)

The maps with grid cell size of 5 m generated from 5 and 10 pulses m^−2^ illustrated the broad similarities of AGC patterns across landscapes (Fig. 8). AGC stock was greatest in plantations with advanced ages (e.g. F634). AGC was more variable in the young plantations (such as F986 and F987). While the site F987 (Fig. 8a1, a2) showed relatively large variability in AGC, the map prediction was not affected by the LiDAR pulse density. Due to the large area and distance between the plantations, the LiDAR data at 5 and 10 pulses m^−2^ at the same site were not collected on the same day. At the time of the first survey to collect LiDAR data with 5 pulses m^−2^, a small stand, not studied herein, 8 years old at site F987 was not completely harvested, as indicated by the red spot in Fig. 8a1. On the other hand, the red spot was not detected in Fig. 8a2, because 1 week later, at the time of the second survey to collect the LiDAR of 10 pulse m^−2^, the small stand was completely harvested.

Although the cost of LiDAR data acquisition was not a central objective that we evaluated in this study, it is nonetheless an important factor to consider. As already mentioned, pulse density has a strong influence on the acquisition cost of LiDAR data, and even though the cost for using LiDAR with high or low pulse density might be lower than the cost of a conventional inventory for AGC in fast-growing Eucalyptus plantations, as presented in [32], it will still be highly expensive for a large area. Although field-based carbon estimations remain necessary for these purposes, integrating LiDAR remote sensing into carbon inventory schemes allows recovery of spatially-explicit AGC estimates across landscapes, while reducing the total costs and need for extensive field-based sampling [31]. In a conventional inventory, normally the variability of the forest attributes at stand level is not always known and therefore less studied [63]. In this study we mapped AGC at the landscape level with a spatial resolution of 5 m, so that it is now possible to compute and capture the variability of AGC at the stand level as well. Moreover, the estimates of AGC in the stands are helpful to determine how much carbon will be stored as forest products made from the timber after harvest.

## Conclusion

The effect of LiDAR pulse density and grid cell size on prediction accuracy of AGC at plot and stand level has been evaluated and analyzed in this paper. First, we found that LiDAR measurements can be used to predict AGC across variable-age Eucalyptus plantations with adequate levels of precision and accuracy using both 5 and 10 pulses m^−2^. Second, we found that H99TH, H10TH, and HVAR metrics were the most important LiDAR metrics for modeling AGC in this study. Third, we found that 5 pulses m^−2^ and grid cell size of 5, 10, 15 and m did not affect the AGC predicted accuracy at plot and stand level. Finally, we demonstrated that the spatial distribution of AGC stocks can be precisely mapped at a grid cell size of 5 m using LiDAR pulse density of 5 pulses m^−2^, which can be used to provide key information for carbon sequestration in this type of ecosystem. Moreover, the promising results for AGC modeling in this study will allow for greater confidence in comparing AGC estimates with varying LiDAR sampling densities for Eucalyptus plantations and assist in decision making towards more cost effective and efficient forest inventory modeling. Although this study presents results for assessing AGC in fast-growing eucalyptus plantations in Brazil, the framework presented herein can serve as a useful methodology, and we hope that the promising results for AGC modeling in this study will stimulate further research and applications not just in Eucalyptus forest plantation in southeast Brazil, but elsewhere as well.

## Abbreviations

AGC: aboveground carbon
CO_2_: carbon dioxide
CNPq: National Council of Technological and Scientific Development
DBH: diameter at breast height
DTM: digital terrain model
FAPESP: State of São Paulo Research Foundation
RF: random forest
RF5: random forest model of 5 pulses m^−2^
RF10: random forest model of 10 pulses m^−2^
GPS: global positioning system
Ht: tree height
LOOCV5: leave-one out cross validation 5 pulses m^−2^
LOOCV10: leave-one out cross validation 5 pulses m^−2^
PVE: percentage of the variance explained
RMSE: root mean squared errors
USFS: US Forest Service
3D: tree-dimensional
