# Erratum to: Combined effect of pulse density and grid cell size on predicting and mapping aboveground carbon in fast-growing Eucalyptus forest plantation using airborne LiDAR data

**DOI:** 10.1186/s13021-017-0082-0

**Published:** 2017-06-30

**Authors:** Carlos Alberto Silva, Andrew Thomas Hudak, Carine Klauberg, Lee Alexandre Vierling, Carlos Gonzalez-Benecke, Samuel de Padua Chaves Carvalho, Luiz Carlos Estraviz Rodriguez, Adrián Cardil

**Affiliations:** 1 Department of Natural Resources and Society, College of Natural Resources, University of Idaho, (UI), 875 Perimeter Drive, Moscow, ID 83843 USA; 2 US Forest Service (USDA), Rocky Mountain Research Station, RMRS, 1221 South Main Street, Moscow, ID 83843 USA; 3 Department of Forest Engineering, Oregon State University, 269 Peavy Hall, Corvallis, OR 97331 USA; 4 College of Forestry, Federal University of Mato Grosso, Av. Fernando Correa da Costa, 2367, Boa Esperança, Cuiabá, MT 78060-900 Brazil; 5 Department of Forest Sciences, College of Agriculture Luiz de Queiroz (ESALQ), University of Sao Paulo (USP), Av. Pádua Dias, 11, Piracicaba, SP 13418-900 Brazil; 6 Tecnosylva, Parque Tecnológico de León, 24009 León, Spain

## Erratum to: Carbon Balance Manage (2017) 12:13 DOI 10.1186/s13021-017-0081-1

Upon publication of the original article [1], the authors noticed that Figure 1 had accidentally been changed to Figure 5. Please see the correct Fig. 1 in this erratum.

**Fig. 1:**
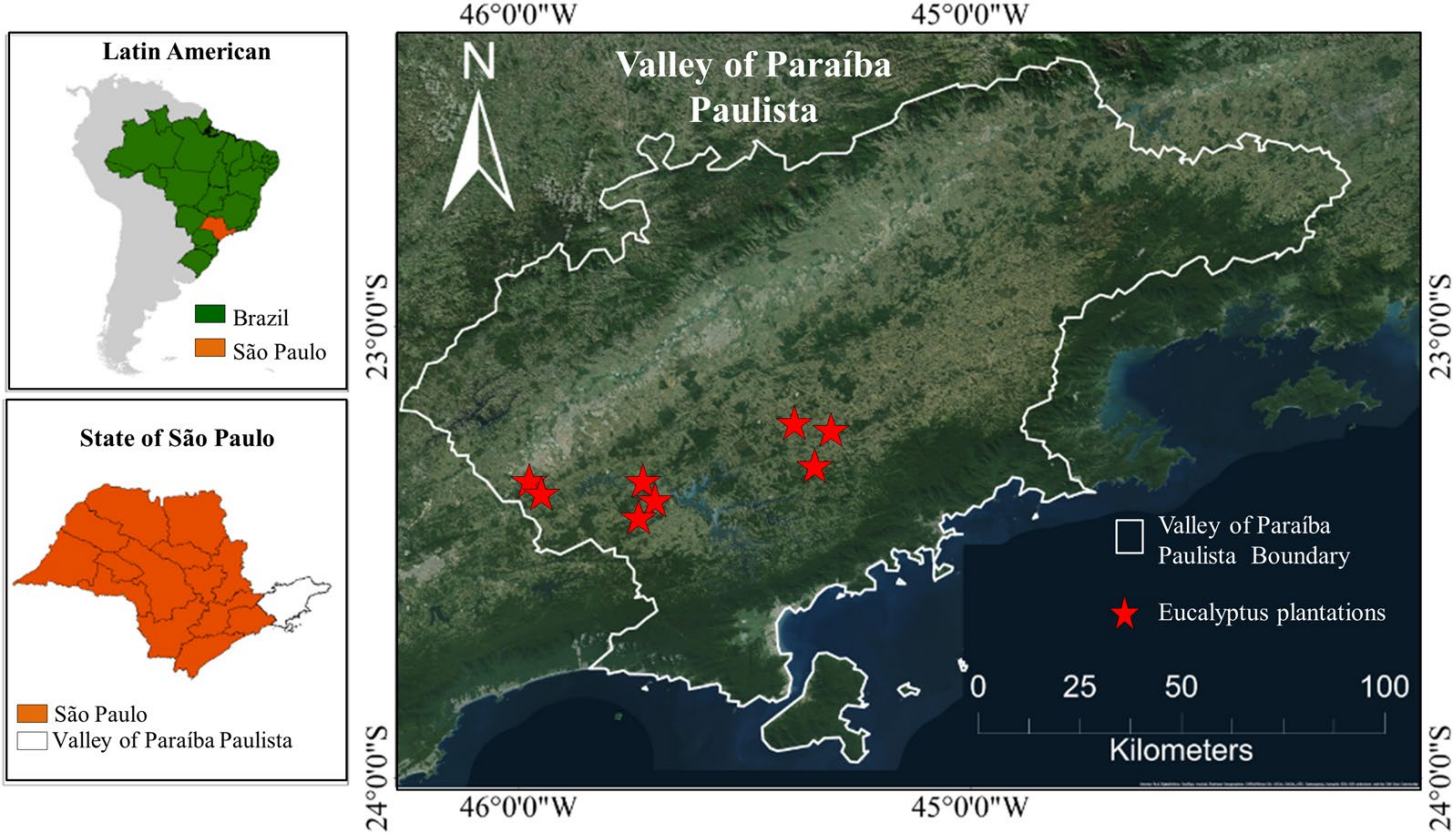
Location of the study area in the State of São Paulo, Brazil. The *stars* indicate the location of the Eucalyptus plantations

This has also been updated in the original article. We apologise for the error made.
